# Assessment of the Quality, Content, and Reliability of YouTube Videos on Automated External Defibrillator Use: A Cross-Sectional Study

**DOI:** 10.1155/emmi/2582984

**Published:** 2025-04-10

**Authors:** Mohamed Fayed, Zeinab Mostafa, Fouzia Ahmed, Kaleem Basharat, Mohammed Adly, Serdar Karakullukçu, Sinan Paslı, Salah Idris, Esam Jerjawi, Keebat Khan

**Affiliations:** ^1^Department of Emergency Medicine, School of Medicine, Hamad Medical Corporation, Doha, Qatar; ^2^Department of Public Health, School of Medicine, Karadeniz Technical University, Trabzon, Turkey; ^3^Department of Emergency Medicine, School of Medicine, Karadeniz Technical University, Trabzon, Turkey

**Keywords:** AED, basic life support, cardiac arrest, defibrillator, resuscitation

## Abstract

**Objective:** The aim of our study was to evaluate the accuracy and reliability of videos available on YouTube and Google showing the use of automated external defibrillators.

**Methods:** Videos available on YouTube and Google between 2020 and 2023 were searched using the search terms “Defibrillator,” “Resuscitation,” “Basic life support,” “Cardiac arrest,” “CPR,” “Cardiac shock,” “Chest trust,” or “First aid.” Data such as the year the video was uploaded, number of views, and video length were collected. The videos were watched and evaluated by two independent emergency physicians. According to the 6-stage evaluation criteria, 1 point was given if the information given in the video was correct and 0 point was given if no information was given. The maximum score was determined as 6 and the minimum score as 1.

**Results:** Out of a total of 315 videos uploaded to the specified platforms, 29 met the inclusion criteria. After the evaluation, the average score given to the videos was 5.45 ± 1.02. When the videos were categorized as low and medium-high according to their fidelity levels, there was no statistically significant difference between these two groups in terms of the number of views, video length, and the score given (*p*=0.469, 0.078, and 0.110, respectively). Videos from institutions were shorter, with a median length of 180 s compared to 289 s for noninstitution uploads (*p*=0.047). Both groups received similar scores, with a median of 6 for each (*p*=0.257).

**Conclusion:** The main findings of our study were that most of the videos were uploaded by health institutions and were shorter. Video scores did not differ according to the level of loyalty of the mannequins used and the uploading source.

## 1. Introduction

YouTube, one of the most popular video-sharing sites in the world, has over 2.1 billion users as of 2022, with videos watched for over a billion hours every day and over 500 h of video uploaded every minute [1]. With such a high number of users and content volume, YouTube has the potential to be an effective educational tool [2, 3]. Health professionals, students, and the general public often use YouTube videos to learn about medical procedures and health education. However, there are serious concerns regarding the accuracy and reliability of these videos. Research indicates that the quality and accuracy of information in videos, especially those covering medical emergencies and critical care, vary significantly [4].

Out-of-hospital cardiac arrest (OHCA) is one of the leading causes of death in Europe and the United States [5, 6]. In cases of cardiac arrest, early recognition, initiation of high-quality chest compressions, and early defibrillation using an automated external defibrillator (AED) are crucial for increasing patient survival [7]. AEDs are portable devices that deliver an electric shock through the chest to restore normal heart rhythm when an abnormal rhythm is detected, significantly increasing the chances of survival when used correctly. AEDs are designed to be used by lay rescuers and are easy to use, with a high accuracy rate in determining the need for a shock [8]. However, sufficient training and knowledge are required to use an AED correctly and effectively. CPR and AED training are provided by organizations such as the American Heart Association, and these devices can be used by anyone to intervene in emergencies [9].

Video-sharing platforms like YouTube are significant tools for providing education on AED use. However, there is insufficient research on the quality, accuracy, and educational value of the videos available on these platforms. Medical videos uploaded are not limited to healthcare professionals; many nonprofessional organizations also publish videos. This can lead to concerns about the reliability and accuracy of the content, potentially resulting in misinformation [10]. This study aims to evaluate the quality and accuracy of educational videos on AED use accessible via YouTube and Google. This study will allow us to examine the potential and reliability of the content available on YouTube's online platform for educational purposes.

## 2. Methods

In this study, the videos to be included in the evaluation were searched using the terms “AED,” “Defibrillator,” “Resuscitation,” “Basic life support,” “Cardiac arrest,” “CPR,” “Cardiac shock,” “Chest thrust,” or “First aid.” The search was conducted for the period from 2020 to 2023 and included only videos uploaded in English. YouTube and Google search engines were used to find the videos.

Data such as the year the video was uploaded, the number of views, and the length of the video were collected. The videos were watched and evaluated by two independent emergency physicians. In case of any disagreement, the content was analyzed by a third physician to reach a final decision.

According to the established criteria, if the information given in the video was correct, 1 point was awarded; if no information was given, 0 points were awarded. The maximum score was set at 6, and the minimum score was 0. Inclusion and exclusion criteria and evaluation parameters are presented in Table 1. Since each step in the process of AED use is important, the videos were evaluated by awarding one point for each step performed correctly and completely. As there is no universally approved scoring system for assessing AED use, a stepwise assessment approach based on the European Resuscitation Council (ERC) guidelines was adopted in this study [11].

The data were analyzed using the Statistical Package for Social Sciences 25.0 (SPSS Inc., Chicago, IL, USA). Means and medians along with standard deviations were used to report continuous variables, while frequencies were calculated for categorical variables. The Kruskal–Wallis test was used for group analysis of numerical variables, and the Mann–Whitney *U* test was used for categorical variables.

## 3. Results

We describe the results of our review of the instructional videos on YouTube about AED use. The total number of videos uploaded between 2020 and 2023 was 146, and 140 were uploaded before 2020. 31 out of 317 reviewed videos met the inclusion criteria. Approximately half of the videos, 50% (*n* = 140), were uploaded prior to 2020 (Table 2).

Most of the videos were uploaded in 2020 and 2022, with 27.6% (*n* = 8) and 31% (*n* = 9), respectively. The remaining videos were uploaded between 2021 and 2023, accounting for 41.3% (*n* = 12). Our findings show that most videos had low-fidelity demonstrations, making up 75.9% (*n* = 22). The videos demonstrating AED use on high-fidelity mannequins were only 17.2% (*n* = 5). As per the findings, the majority of videos (72.4%, *n* = 21) were uploaded by institutions, while 27.6% (*n* = 8) were uploaded by individuals not affiliated with any institution. Further analysis of the data reveals that the average views ranged from a minimum of 13 to a maximum of 800,788 (mean = 54,538.4). The videos had a mean length of 277.2 s, with the shortest being only 59 s. The mean score from a total of 6 scores was 5.45 (Table 3). Upon comparison, it was observed that the average views did not differ significantly between the low and medium-high fidelity levels, with median values of 11,762.5 and 14,908, respectively, indicating no significant difference between the two levels (*p*=0.469). Additionally, no statistical difference was observed between the videos' length for the low and medium-high levels of fidelity (*p*=0.078). The videos demonstrated on the medium-high level of fidelity had a higher median score of 6, as compared to the median score of 2 observed with low-fidelity mannequins. However, no statistical difference was found between the median scores of both (*p*=0.110) (Table 4).

The average views did not differ depending on whether uploaded by medical organizations or personnel without medical background (*p*=0.943). The videos uploaded by the institutions had a lower length of the videos with a median of 180 as compared to the median of 289 uploaded by noninstitutions (*p*=0.047). There was no statistical difference noted in terms of scores received by the source of upload with a median of 6 for both (*p*=0.257) (Table 5).

## 4. Discussion

Social media's popularity is not limited to entertainment; it has also been widely used to share knowledge. Many instructional videos are posted on YouTube, including healthcare-related content. The reliability of these videos is questionable, as there are no regulations on the accuracy of the content presented. We aimed to assess the quality of AED videos uploaded on YouTube. Currently, there is no universally validated tool specifically designed for assessing the quality of AED use. Therefore, we used a scoring system based on the key steps outlined in the ERC guidelines, which emphasize the critical components of AED use [11]. Each step—scene safety, device activation, electrode pad placement, rhythm analysis, shock delivery, and post-shock actions—was assigned “one point” to ensure a structured and objective evaluation. This approach ensures that the evaluation reflects established best practices in AED training. While our method is not a standardized universal scoring system, it provides a structured and reproducible framework for assessing video quality. In our study, the key findings were that most of the videos were uploaded by medical institutions, and they were shorter. However, there was no difference in scores compared to noninstitutional sources. Our study revealed that the majority of the videos demonstrating the use of AED were performed using low-fidelity mannequins and received low scores overall. Conversely, videos featuring medium to high levels of fidelity scored higher compared to the established standard. Our findings of the study align with the studies previously done, highlighting the variation in quality and reliability of the medical-related content on YouTube. We will discuss the issues identified and factors related to them.

The level of fidelity has an essential impact on the teaching during the simulation. As we know from previous studies, high-fidelity simulations are associated with better learning outcomes [12]. Our findings contribute to this by indicating that although videos utilizing high-fidelity mannequins received higher median scores, the difference was not statistically significant. This suggests that adherence to guidelines may be more critical than fidelity level alone. A study by Wayne et al. demonstrated that simulation-based programs improve the learning outcomes [13]. On the other hand, the study by Bruce et al. showed that high-fidelity simulation resulted in better knowledge retention but there was no difference after a follow-up of 1 year [14]. Similarly, Massoth et al. found out that the high-fidelity simulation is not necessarily better than the other [15]. These findings suggest that while high-fidelity simulations may enhance learning, their impact on educational effectiveness in publicly available videos remains unclear. Given the importance of accuracy in medical education, further research is needed to determine the optimal balance between fidelity and accessibility. Given that AED use is designed for both medical professionals and laypersons, ensuring the accuracy and completeness of educational videos is critical.

Our research indicates that videos uploaded by institutions were significantly shorter in duration compared to those uploaded by noninstitutions; however, both types received similar scores, despite the wide variation in video durations. In comparison to our study, Hawryluk et al. observed that the high-quality videos had longer duration and higher number of views also. The mean duration of the videos was 375 s in their study [16]. Similarly, the study by Hsuen et al. showed that the videos with high quality had longer duration and received a higher number of views as well [17]. On the other hand, the average length of videos reported by Vilela et al. was 150 s, indicating that most videos were of short duration. Like our study, it was found that videos of variable lengths ranged from a few seconds to several minutes. While some studies have reported a positive correlation between video duration and quality, our study indicates that duration alone may not be a definitive predictor of quality. As long as the fundamental and guideline-based essential information is provided, there may not be necessity for longer durations.

In our study, the videos were mostly uploaded by institutions. It is interesting to note that the scores did not differ whether the videos were uploaded by the institutions or nonprofessional personnel. This finding suggests that in cases where the subject matter involves standardized and widely taught procedures such as AED use, the accuracy of the content may be less dependent on the uploader's professional background and more on adherence to established guidelines. Therefore, rather than focusing solely on the source of the videos, future efforts should prioritize the implementation of quality control mechanisms that assess and certify the accuracy of online educational content. According to the study by Vilela et al., there was no video that achieved complete compliance with the standard guidelines even though most of the videos (40.5%) were uploaded by healthcare professionals [18]. Similarly, a study by Ertem et al. and Gurler et al. showed that the majority of the videos (87% and 70%, respectively) were uploaded by professionals [19, 20]. Additionally, Ertem et al. found that videos produced by healthcare professionals were of higher quality compared to those created by non-healthcare professionals. Similar findings were noted by Okagbue et al. and Hawryluk et al., where the videos uploaded by medical professionals and institutions were more reliable, had higher mean scores, and were less likely to share incorrect information [16, 21]. A systematic review by Osman et al. found that professional sources provide more reliable content and facilitate users' access to accurate information [22]. Similarly, in a study evaluating the reliability and quality of YouTube videos used in the education of surgical residents, videos prepared by physicians were considered more reliable [23]. Though past studies show good reliability of the videos uploaded by the professionals as compared to noninstitutional uploads, our study showed that there was no difference between either of these.

In our study, the average views were similar and did not depend on the videos by professionals or noninstitutional personnel. On the contrary, Hawryluk et al. noted that videos uploaded by physicians had longer length of duration and received maximum number of views as well. Similarly, Okagbue et al. reported that views were higher for the videos by professionals comparatively. Gurler et al. and Okagbue et al. found that videos that were of low quality were viewed more frequently [20, 21]. Health-related content on YouTube is often popularity-driven, which can be a breeding ground for the spread of misinformation [22]. Indeed, the fact that low-quality videos receive more views and engagement reveals the lack of a regulatory framework for content quality. As noted in Joshi et al.'s study, guidance from medical professionals is necessary to access accurate information [24]. Developing algorithms that prioritize expert-reviewed content could help mitigate the risk of misleading instructional materials being widely disseminated. To improve the reliability of educational videos, collaboration between healthcare organizations, regulatory bodies, and digital platforms is essential. Implementing expert validation processes and ranking systems based on accuracy could significantly enhance the quality of publicly available health education content.

### 4.1. Limitations

There are some potential limitations in the study. First, we only selected videos uploaded in English, which excludes a large number of videos from non-English-speaking countries. Second, YouTube is a dynamic platform where videos are frequently uploaded and viewed. Due to our examination of the videos within a limited time period, there may have been changes in the viewing parameters that could have resulted in different outcomes. As there is no universally validated tool for assessing AED instructional videos, this study utilized a structured scoring system based on ERC guidelines, which, while systematic, has not undergone formal validation.

## 5. Conclusion

Our study has shed light on the quality and reliability of AED videos uploaded on YouTube. By addressing the issues identified, we can enhance the credibility of medical-related content on the platform. This, in turn, will help the audience learn educational content more reliably. Overall, it is important to improve the quality and accuracy of health-related information on YouTube to deliver authentic content.

## Figures and Tables

**Table 1:** Criteria for inclusion/exclusion and evaluation standards.

**Inclusion criteria**

Educational videos on AED use from 2020 up-to-date
AED term, automated electric defibrillator, cardiac arrest, chest thrust
Videos which were conducted in English language only
Videos for AED use on human and nonhuman subject (e.g., mannequins)

**Exclusion criteria**

All videos which were conducted in non-English language
All educational AED videos which were uploaded before 2020
Noneducational videos (e.g., promo, commercial, comics, and news)
Videos which contain animal training for AED use
Videos which demonstrate manual electric cardioversion

**Contents for evaluation: (score from 0–6)**

1. Indication of AED use
(Recognition of cardiac arrest, checking pulse, identification of respiratory arrest, calling for help, giving mouth to mouth breath if possible, starting CPR till arrival of EMS or till defibrillator is available)
2. Turning on the AED
(Open the AED device, set it up, attach cables to adhesive pads if not readily attached, call 999)
3. Application of pads on the chest
(Evaluation of the steps of pad application, e.g., chest wall exposure, ensure skin dryness, removal of any medical patches, e.g., nicotine patch if any, correct pad placement according to the demonstrated diagrams)
4. Follow the instructions
(Pressing the analyze button on the machine and follow the voice)
5. Stand back, deliver the shock
(Stepping back to ensure safety, pushing the button in case the shock is required)
6. CPR performance
(Checking the response after shock delivery, keeping patient in recovery position, or perform CPR if not recovered till first responder arrives)
• Score is graded from 0 to 6 according to the previously set criteria
• Correct information was scored as 1 and incorrect or missing information was scored as 0

**Table 2:** Excluded video content.

Excluded content	*n*	%
Uploaded before 2020	140	49.0
Commercial	75	26.2
Non-English language	29	10.1
Manuel AED	16	5.6
Pediatrics	10	3.5
Animal training	9	3.1
Comics	4	1.4
AED maintenance	3	1.1
Total	286	100.0

**Table 3:** Characteristics of the included videos.

Year of uploading	*n*	%
2020	8	27.6
2021	7	24.1
2022	9	31.0
2023	5	17.2

**Level of fidelity**		

Low	22	75.9
Medium	2	6.9
High	5	17.2

**Source of the videos**		

Institution	21	72.4
Noninstitution	8	27.6

**Video metrics and attributes**	**Mean + std**	**Median (min**-**max)**

Average views	54,538 ± 149,210	13,811 (13–800,788)
Length (second)	277 ± 221	191 (59–1020)
Score	5.45 ± 1.02	6 (2–6)

**Table 4:** Number of views, length, and scores of videos according to their fidelity.

Videos		Low fidelity	Medium-high fidelity	*p* ^∗^
Average views	Mean ± std	65,028 ± 170,740	21,572 ± 13,884	0.469
	Median (min-max)	11,762 (13–800,778)	14,908 (8210–44,611)	

Length (second)	Mean ± std	246 ± 192	376 ± 290	0.078
	Median (min-max)	183 (59–840)	265 (166–1020)	

Scores	Mean ± std	5.27 ± 1.12	6	0.110
	Median (min-max)	2 (2–6)	6 (6-6)	

*Note:* A *p* value of < 0.05 was considered statistically significant.

^∗^Mann–Whitney *U* test.

**Table 5:** Average views, length of the videos, and scores based on the source of upload.

Source of the upload		Institution	Noninstitution	*p* ^∗^
Average views	Mean ± std	28,022 ± 40,109	124,143 ± 277,149	0.943
	Median (min-max)	12,525 (13–139,495)	14,359 (22–800,778)	

Length of the video (second)	Mean ± std	235 ± 186	387 ± 278	0.047
	Median (min-max)	180 (59–840)	289 (156–1020)	

Scores	Mean ± std	5.29 ± 1.15	5.88 ± 0.35	0.257
	Median (min-max)	6 (2–6)	6 (5-6)	

^∗^Mann–Whitney *U* test.

## Data Availability

The data that support the findings of this study are available on request from the corresponding author. The data are not publicly available due to privacy or ethical restrictions.
